# Polydatin radiosensitizes lung cancer while preventing radiation injuries by modulating tumor-infiltrating B cells

**DOI:** 10.1007/s00432-023-04762-7

**Published:** 2023-05-23

**Authors:** Jiaming Guo, Wen Ding, Shanlin Cai, Pan Ren, Fengxu Chen, Jiawen Wang, Kai Fang, Bailong Li, Jianming Cai

**Affiliations:** 1 School of Public Health and Management, Wenzhou Medical University, Wenzhou, 325035 China; 2 Department of Radiation Medicine, College of Naval Medicine, Naval Medical University, Shanghai, 200433 China; 3 Department of Medicine College, Jiangnan University, Wuxi, 214000 Jiangsu China; 4 The 929th Navy Hospital, Naval Medical University, Shanghai, 200433 China; 5 School of Basic Medicine, Naval Medical University, Shanghai, 200433 China

**Keywords:** Lung cancer, Radiation sensitization, Radioprotection, Polydatin, Immunomodulation, Tumor microenvironment

## Abstract

**Background:**

Acquired radio-resistance and the undesired normal tissue radiation injuries seriously discount the therapeutic effect of lung cancer radiotherapy. In this study, we aimed to explore the role and potential mechanism of polydatin in simultaneously decreasing radioresistance and radiation injuries.

**Methods:**

The tumor-bearing model of nude mice was used to investigate the tumor inhibition of polydatin on lung cancer and its effect on radiosensitivity, and the effect of polydatin on B cell infiltration in cancerous tissue was investigated. In addition, we performed systemic radiotherapy on BABL/C mice and evaluated the protective effect of polydatin on radiation injury by the Kaplan–Meier survival curve. Moreover, the regulation of polydatin on proliferation and apoptosis of A549 cells was also investigated in vitro.

**Results:**

In this study, it is first found that polydatin inhibits the growth and promotes the radiosensitivity of lung cancer while reducing the radiation damage of the healthy tissue. Further, it is evidenced that the major mechanism relies on its regulation on body’s immune function, and in particular, the inhibition of radiation-induced B cell infiltration in tumor tissue.

**Conclusion:**

These findings show that in addition to tumor inhibition, polydatin also promotes the sensitivity and reduces the adverse reactions of radiotherapy, making itself a promising candidate for boosting lung cancer radiotherapy efficacy.

**Supplementary Information:**

The online version contains supplementary material available at 10.1007/s00432-023-04762-7.

## Introduction

Lung cancer is the leading cause of cancer-related deaths worldwide, and research to improve treatment effectiveness has been one of the hottest topics (Thai et al. 2021). Great advances have been made in the treatment of lung cancer, including routine surgical treatment, chemotherapy, immunotherapy, and radiotherapy. Among them, radiotherapy plays an increasingly important role, with more than 70% of lung cancer patients receiving radiotherapy during the whole treatment process (Delaney et al. 2003). However, the development of acquired radio-resistance during lung cancer radiotherapy compromises the curative outcomes seriously. Thus, steps attempting to enhance the radiosensitivity of lung tumors haven’t stopped for decades.

Apparently, the ideal lung tumor radio-sensitizer should sensitize the cancer cells to radiation while processing little toxicity to normal tissues, or instead, conferring protective effects against radiation side effects. Thus, scientists have been trying to seek a series of radio-modifiers with radio-sensitizing and -protective dual functional benefits (abbreviated as RMDF), most of which were theoretically based on the differentially regulated singling pathways according to the variance between tumor and normal tissue genetic background by the same administrated reagent (Miyake et al. 2019; Anjaly and Tiku 2022). However, other mechanistic strategy-supported radio-modifiers may exert even better efficacy during lung radiotherapy, which attracts our study interest greatly.

Recently, we found that polydatin (also known as piceid or 3,4′,5-trihydroxystilbene-3-*O*-β-d-glucoside, PD), the glycoside of resveratrol, holds the potential to be one of the RMDFs. PD is a crystal compound extracted from *Polygonum cuspidatum Sieb. et Zucc* (a traditional Chinese medicine widely used clinically all over China), and it can also be isolated in many other plants, such as hop pellets, grape, peanut, hop cones, red wines, cocoa, or chocolate products (Mikulski and Molski 2010). It has been soundly evidenced that PD exerts extensive bioactive effects such as correction of oxidative stress (Li et al. 2013; Huang et al. 2015), alleviating inflammation (Shiyu et al. 2011), suppressing allergy (Yang et al. 2013), mitigating metabolic diseases, etc. (Du et al. 2009; Miao et al. 2012). Specifically, a collection of studies has reported that PD can directly suppress the proliferation and migration of different lung cancer cell lines via its regulatory effects on inflammation, DNA damage response, cell cycle arrest, senescence, and apoptosis (Verma and Tiku 2022; Zhang et al. 2014; Zou et al. 2018). Moreover, in our previous studies, we observed that PD could exhibit excellent radioprotective effects against systemic radiation injury containing lung damage through regulating immune and anti-oxidation function (Guo et al. 2020; Cao et al. 2017). However, it is not clear whether PD can be used as a radiosensitizer to cancer tissue in lung cancer patients at the same time as a radioprotector for the radiation injuries of the normal tissue (mitigating the side effects of radiation therapy).

Based on the published knowledge and our previous work, we hypothesize that PD is potentially a novel RMDF to be applied during lung cancer radiotherapy, the mechanism of which involves elevating radiation-induced cancer cell apoptosis and regulation on the immune system. In the present study, the radio-sensitization of PD on lung cancer was examined both in vitro and in vivo, and the radioprotection of PD was re-confirmed again. Furthermore, the mechanism for PD’s RMDF regulatory effects on lung cancer radiotherapy was explored as well.

## Materials and methods

### Subcutaneous tumor-bearing nude mice model

Thirty-two Balb/c nude mice (male 5–6 weeks) were purchased from Shanghai Laboratory Animal Center of Chinese Academy of Science. All living conditions and protocols were approved by the Naval Medical University Institutional Animal Care and Use Committee in accordance with the Guide for Care and Use of Laboratory Animals published by the US NIH (publication no. 96-01). After anesthesia of nude mice with isoflurane, A549-luciferase cells (2 × 10^6^) were subcutaneously injected into the right axilla of nude mice. Tumor size was monitored every 3 days using calipers. Tumor volume (mm^3^) was calculated as *V* = *a* × *b* × *c*/2, where a represents the long axis diameter, *b* indicates the width axis diameter, and *c* was the depth axis diameter. Tumors were grown in mice fed regular chow until they had reached a mean volume of ~ 200 mm^3^. At this time, mice were sequentially divided into 4 groups (Con group, PD group, IR group, and IR + PD group; 8 mice for each group) according to the tumor volume. PD and dimethyl sulfoxide (DMSO) were purchased from Sigma (USA). PD was dissolved in DMSO to prepare the PD working solution (20 mg/ml). The PD and IR + PD groups were administered orally with the PD working solution (20 mg/ml, 200 μl), and the con and IR groups with DMSO (200 μl) once per day for 7 days. Then, IR at the dose of 20 Gy (dose rate: 1 Gy/min) was delivered locally to the tumor sites of each mouse all at once two days after oral administration. Any group of tumors that reached approximately 1000 mm^3^ in volume or that had been observed for 8 weeks were then sacrificed by cervical dislocation and their tumor tissue was harvested for the following examination.

### In vivo imaging monitoring subcutaneous tumor growth

Subcutaneous tumor-bearing nude mice were intraperitoneally injected with d-luciferin potassium salt (S31462, Shanghai Yuanye Bio-Technology Co., Ltd) and imaged by In-Vivo Imaging System (QuickView 3000, LABATECH GmbH. Bergstrasse 14 A-5020, Salzburg, Austria) which was performed 15 min after injection. In vivo imaging was performed before treatments and once again two weeks after treatments. The photon signaling intensity of each time point was recorded in each treatment group.

### Flow cytometry detecting tumor-associated B cells

Tumor tissue specimens were processed to single cell suspensions using mechanical and enzymatic digestion (320 U/ml Collagenase IV, Worthington, and 100 U/ml DNAse I, Applichem). Samples were then filtered through 40 μm strainers, and cells were fixed with 1% paraformaldehyde (BarNoar) diluted in FACS buffer for 20 min at 4 °C. Cells were then washed with FACS buffer, centrifuged at 1200 rpm for 5 min at 4 °C, and re-suspended in FACS buffer. Subsequently, 1 × 10^5^ cells in 100 μl were stained for surface markers with the following antibodies: PE-conjugated CD19 (Biogems), APC-conjugated B220 (Biogems), and FITC-conjugated rabbit IgM (Biogems). Antibodies were diluted in FACS buffer according to the manufacturers' instruction and incubated with cells for 30 min at room temperature away from light. Cells were then diluted to proper volume with additional FACS buffer, and cell staining was evaluated using Flow Cytometer (CytoFlex, Beckman Coulter). Data were analyzed using FlowJo software. Intra-tumoral B cells were identified as B220^+^ or CD19^+^.

### Irradiation

^60^Co-gamma rays in the radiation center of Naval Medical University were used to perform irradiation exposure at room temperature. Mice and cells (with or without PD pretreatment) were exposed to different doses of IR, depending upon the treatment requirement.

### Animal survival

Twenty-four BABL/c mice (male, 8 weeks) were purchased from Shanghai Laboratory Animal Center of Chinese Academy of Science. All living conditions and protocols were approved by the Naval Medical University Institutional Animal Care and Use Committee in accordance with the Guide for Care and Use of Laboratory Animals published by the US NIH (publication no. 96-01). Mice were randomly divided into three groups (Con group, IR group, and IR + PD group) equally. The IR + PD group was intraperitoneally injected with PD (20 mg/ml, 200 μl) once per day for 7 days and the Con and IR groups with DMSO (5%) 200 μl/mice. Then, mice were exposed at different doses (8 Gy, 9 Gy, 10 Gy, dose rate: 1 Gy/min) once two days after injections. The survival status and general condition of the mice were observed and recorded daily. After 40 days of continuous observation, the remaining mice were sacrificed by the spinal dislocation method.

### Cell culture

The human lung epithelial cell line (BEAS-2B) and lung cancer cell line (A549 or A549-luciferase) were cultured in DMEM medium with 10% (v/v) fetal bovine serum and 1% (v/v) penicillin/streptomycin solution in an incubator with 5% CO_2_ at 37 °C. When the cell density was 70–80%, the subsequent experiments were carried out.

### Peripheral complete blood count analysis

Immediately after the mice were anesthetized by an anesthesia apparatus (Norvap, U.K.) with isoflurane, blood samples (0.7 ml) were obtained from the angular vein and collected into the ethylenediaminetetraacetic acid (EDTA)-coated anticoagulant tubes for the following analysis via an automatic blood cell analyzer (XN-V series Sysmex, Japan) according to the manufacturer's instruction. Then, a comprehensive result involving white blood cells (WBCs), red blood cells (RBCs), platelets (PLTs), and their subsets were outputted and indicators including PLR (platelet-to-lymphocyte ratio), NLR (neutrophil-to-lymphocyte ratio), NMR (neutrophil-to-monocyte ratio), and LMR (lymphocyte-to-monocyte ratio) were also calculated.

### Cell proliferation assay

Cell Counting Kit-8 (CCK-8) test was used for cell proliferation assay. Cells were seeded in a 96-well plate with 1 × 10^4^ cells per well in quintuplicate and treated with PD at different doses (0 μM, 20 μM, 40 μM, 60 μM, 80 μM, 100 μM, and 120 μM). After culturing for 24 h, 48 h, and 72 h, 10 μl of CCK-8 reagents (Beyotime, China) was added and the absorbance at 450 nm was measured after 2 h using a microreader. For combined IR, PD was added to the plates 1 h before 8 Gy γ-ray irradiation exposure, and cell proliferation was measured at 24 h, 48 h, and 72 h. The inhibition rate of cell proliferation was calculated as [1 − (treated cells ÷ untreated cells) × 100%].

### Colony-formation assay and dose–survival curves

Cells were seeded in 6-well plates at different concentrations (200, 250, 500, 1000, and 2000 per well) in triplicate and allowed to incubate for 12 h before irradiation. PD (50 and 100 μM) was added 1 h before irradiation. Cells were respectively exposed to different doses of radiation (2, 4, 6, and 8 Gy). After IR exposure, cells were returned to the incubator for another 12 days until the formed colonies were easily visible by the naked eye. Then, the plates were fixed with 100% ethanol for 10 min, stained with 0.5% Crystal Violet for 20 min, and carefully cleaned with water. The colonies containing more than 50 cells were defined as survivors, and survival fraction (SF) was calculated. The dose–survival curve equations are as follows: plating efficiency (PE) = number of colonies/number of seeded cells × 100% for unirradiated controls; survival fraction (SF) = number of colonies/number of seeded cells × PE × 100%.

### Apoptosis assay

Cells were seeded in six-well plates and treated with PD alone (50 μM), radiation alone (8 Gy), or a combination of both. PD was added 1 h before radiation and 48 h after radiation. Cells were collected and washed twice in cold PBS. Then, cells were re-suspended in 100 μl of 1 × Binding Buffer and incubated with 5 μl of Annexin V-FITC and 5 μl of PI Staining Solution for 15 min. Then, 400 μl of 1 × Binding Buffer was added to plates and samples were detected using a flow cytometer within one hour.

### Immunohistochemistry

Briefly, sections were dewaxed and rehydrated. Antigen retrieval was performed by pretreatment of the slides in citrate buffer (pH 6.0) in a microwave oven for 23 min. The slides were incubated with PBS containing 3% hydrogen peroxide for 25 min, PBS containing 5% BSA for 30 min, and subsequently the primary antibody at 4 °C overnight. After the sections are slightly shaken and dried, the tissues are covered with secondary antibody (HRP labeled) from the corresponding species of primary antibody and incubated at room temperature for 50 min, followed by reaction with diaminobenzidine and counterstaining with Mayer’s hematoxylin. To score each slide, at least eight individual fields were chosen. We calculated the proportion of DAB chromogenic positive using ImageJ. Results are expressed as positive cells percentage.

### HE staining

The harvested tumor tissues were isolated, fixed by 4% paraformaldehyde, embedded in paraffin, and sliced into 4 µm thick sections. The sections were deparaffinized, rehydrated following the standard techniques, and stained with hematoxylin and eosin solution. Observations were made under a light microscope after the sections were dehydrated and cleared with xylene.

### Statistical analysis

Statistical analyses were performed using GraphPad Prism 9.3 software. All quantitative data are presented as the mean ± SEM and were obtained from at least three independent experiments. For normalization, sham control values were set to 100%, and values of other treatment groups are displayed as a percent of those of sham control. Student’s two-tailed unpaired *t* tests were used to compare the differences between two groups. Statistical significance was set at *p* < 0.05.

## Results

### PD inhibits tumor growth and promotes IR sensitivity of A549 cells in vivo

**Fig. 1:**
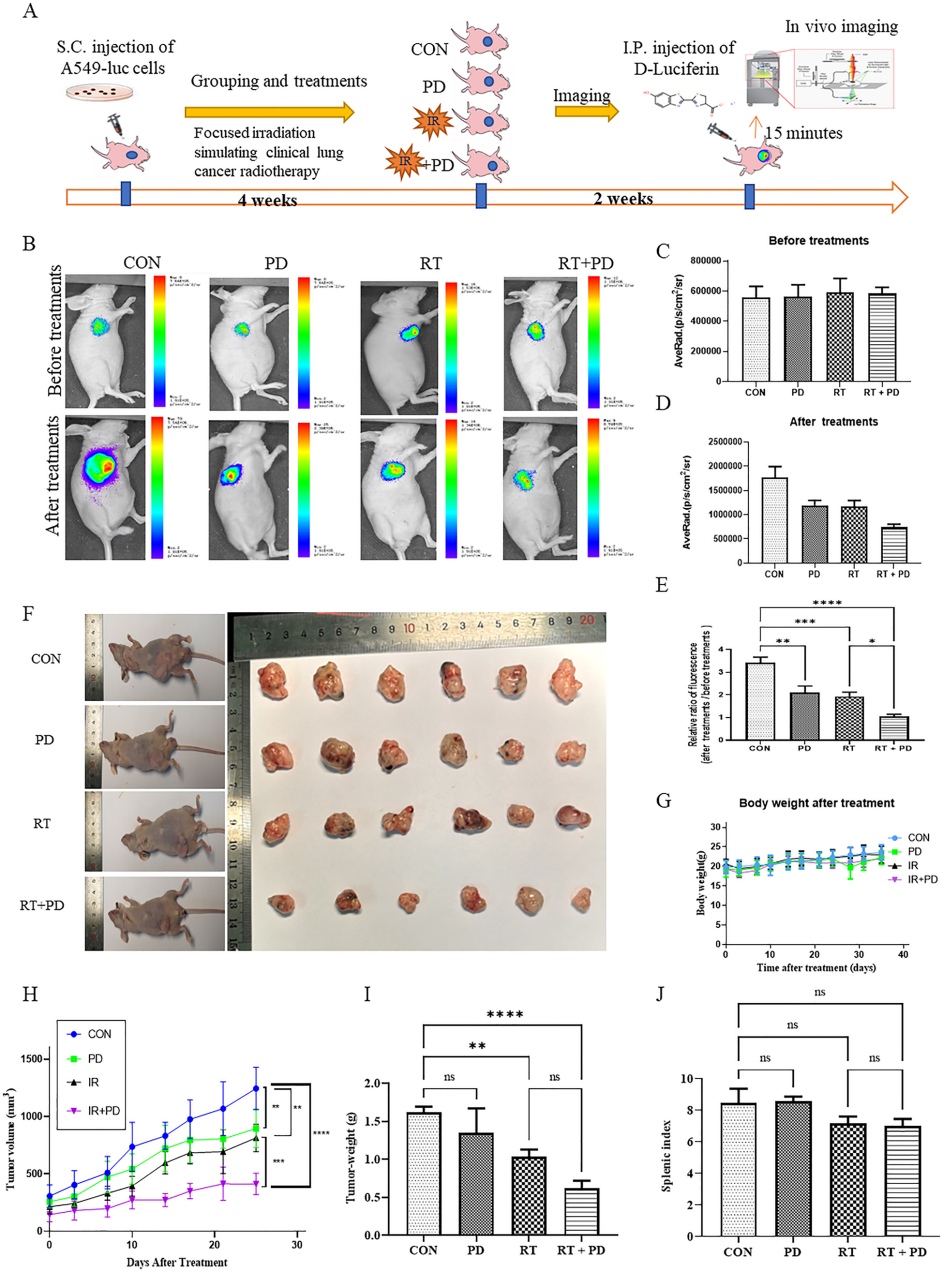
Radiosensitivity of PD against lung cancer in nude mice xenograft model. a Flow chart of tumor-bearing experiment in nude mice. **b** Representative photos of bioluminescence images in different groups. **c**–**e** Quantitative comparison of mean tumor volume in different treatment groups. **f** Representative photos of mice and the dissected tumors from different groups. **g** Mean body weight curves of tumor-bearing mice in different treatment groups. **h** Curves of mean tumor volume in different treatment groups. **i** Quantitative comparison of tumor weight in different treatment groups. **j** Quantitative comparison of the splenic index in different treatment groups. NS represents no significance, *p < 0.05, **p < 0.01, ***p < 0.001

To evaluate whether PD could sensitize lung tumors to IR, we established a mouse xenograft model for lung cancer combined with simulated clinical radiotherapy and examined the tumor growth (Fig. 1a). First, we used the in vivo fluorescent imaging method to measure tumor size. Four weeks after A549 subcutaneous implantation, the bioluminescence images indicated that the fluorescent activity of different groups was similar and the tumor area of each mouse indicated that it was ready for treatments (200 mm^3^ ± 50 mm^3^, Fig. 1b, c). Two weeks after the corresponding treatments (Con, PD, RT, and PD + RT), tumors of every group had grown but to various degrees. Though all of the therapeutic interventions repressed the tumor growth significantly, the PD + IR group managed to enhance the tumor shrinkage markedly in comparison to either the PD- or RT-only group (Fig. 1b, d, e). Additionally, the tumor volume curve fitted by the vernier caliper-measured tumor diameters confirmed this trend again (Fig. 1f, j). As can be seen from Fig. 1f, h, both PD and RT independently decrease the tumor propagation, but in the IR + PD group tumors grew significantly lower than the others. Moreover, this pattern was consistent with the comparison of tumor weight (Fig. 1i). Meanwhile, we did not detect any significant difference among these four treatment groups either regarding body weight or splenic index (Fig. 1g, j). Together, PD exerted a role in tumor suppression and radio-sensitization without adverse impacts in lung carcinoma.

### Radioprotection of PD in a mouse radiation injury model

**Fig. 2:**
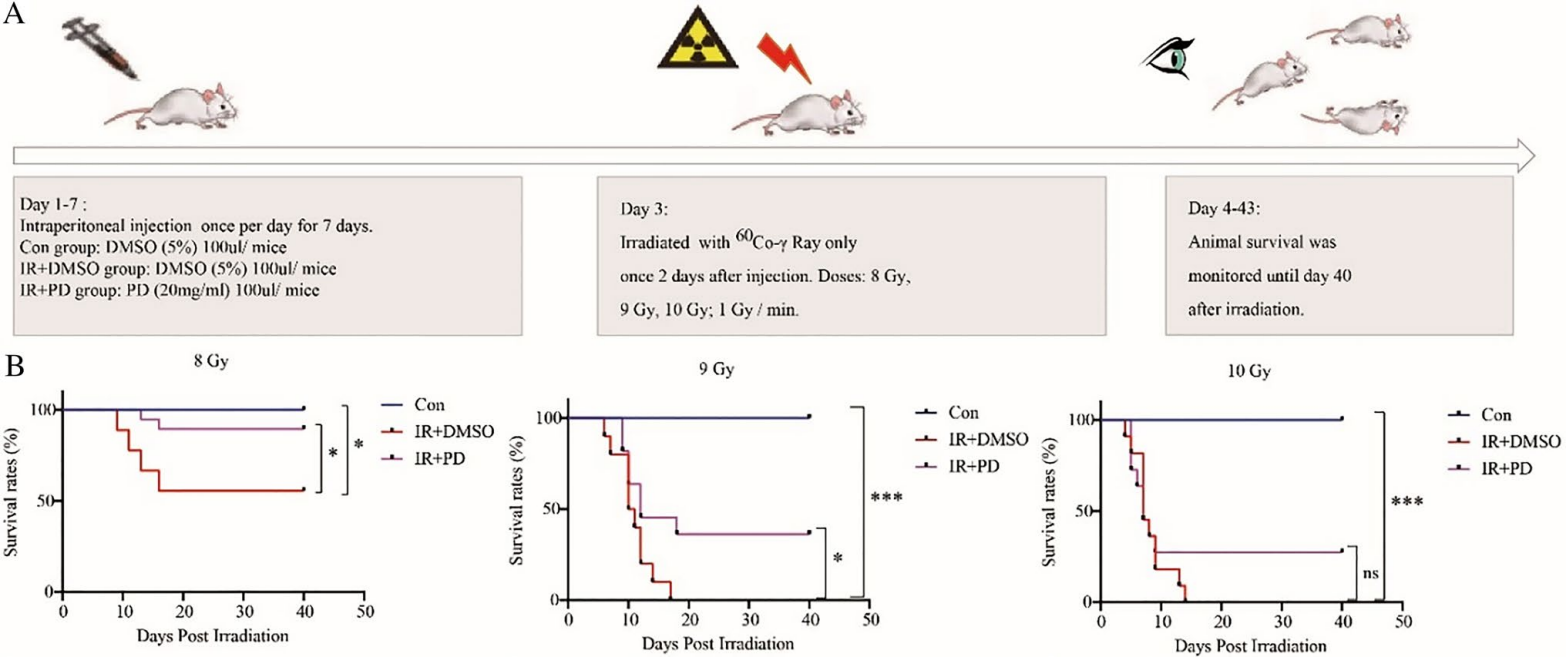
Survival curves of different treatment groups. **a** Design and flow chart of animal survival experiment using Balb/c mice. **b** Balb/c mice were randomly assigned into different groups (10 mice for each group including CON group, IR + DMSO group, and IR + PD group). Administration of DMSO (5%) or PD (2 mg/mice) was performed intraperitoneally 2 days before ^60^Co-γ radiation exposure (A: 8 Gy; B: 9 Gy; C: 10 Gy; dose rate: 1 Gy / min). Animal survival was monitored until day 40 after exposure. *p < 0.05, **p < 0.01, ***p < 0.001, ****p < 0.0001

To evaluate the impact of PD on the adverse radiation injuries of normal tissue when lung cancer radiotherapy is applied, we conducted the animal survival analysis as displayed in Fig. 2a. With the increase of total body irradiation dose (8 Gy, 9 Gy, and 10 Gy), the survival time of mice was shortened (Fig. 2b). Additionally, PD significantly increased the survival probability when they were exposed to lethal doses of 8 Gy or 9 Gy. However, no statistical differences were calculated when the IR doses reached a more serious level of 10 Gy, but the radioprotective trend was still obvious (Fig. 2b). In brief, these data reflect a definite protective effect of PD against IR when animals go through lethal radiation damages, which can be deemed as an extreme scenario of IR injuries when curing tumors including lung cancer.

### Evaluation of the direct regulatory impact of PD on the radiosensitivity of both normal and cancerous lung cells in vitro

**Fig. 3:**
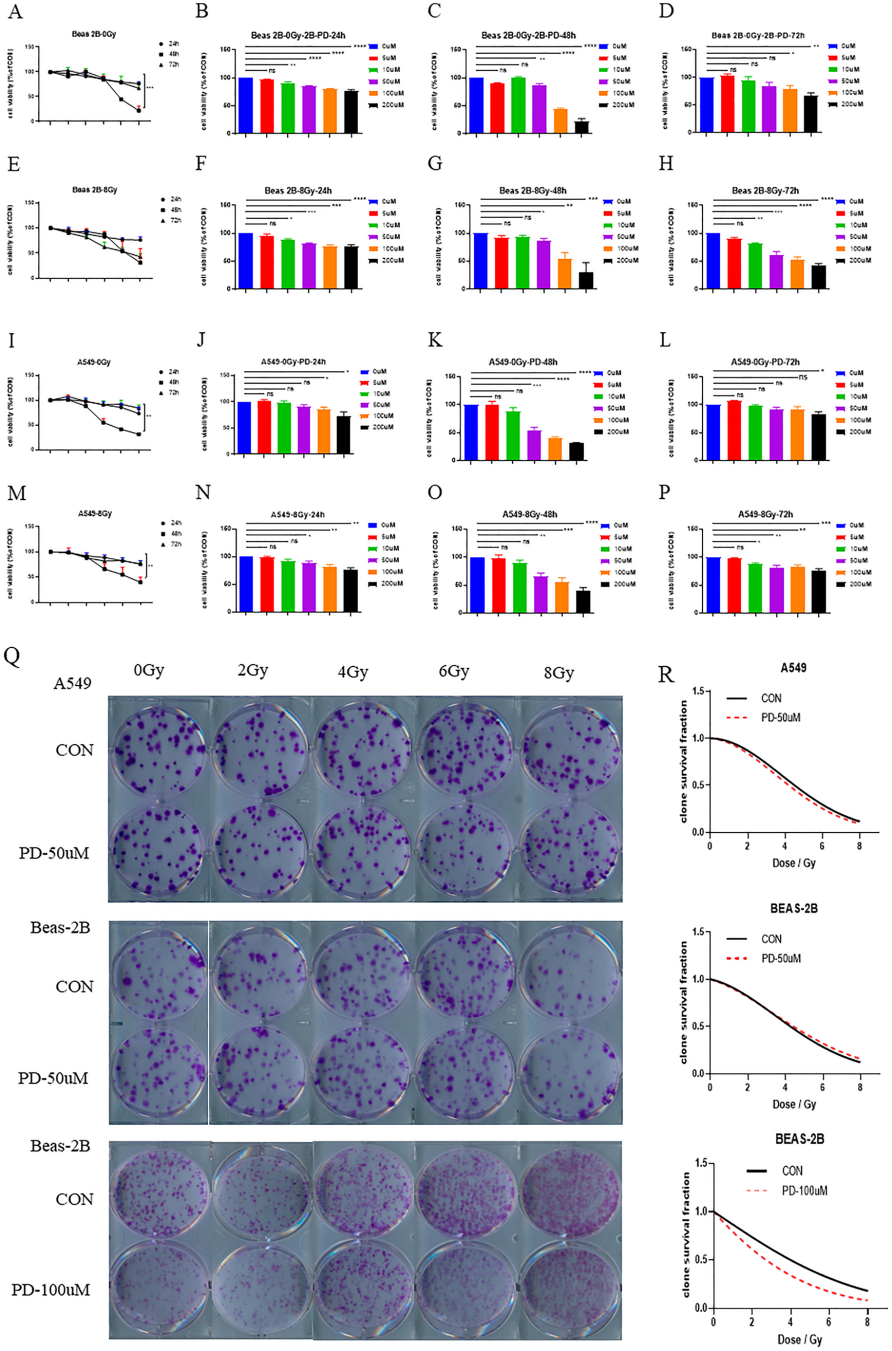
Cell viability of different groups tested by CCK-8 assay. **a**–**p** The CCK-8 assay was used to evaluate PD’s impact on cell viability short periods after treatments. **q** The representative pictures and **r** the fitted clone survival fraction curves of different groups were generated by clonogenic assay. NS represents no significance, *p < 0.05, **p < 0.01, ***p < 0.001, ****p < 0.0001

To explore the possible mechanism of PD regulating the radiation response in vivo, we first used CCK-8 assay to detect the direct effect of PD on cell viability shortly after IR (as long as 72 h). On the one hand, we observed that the viability of either lung epithelial cell line BEAS 2B or lung adenocarcinoma cell line A549 was decreased by PD in a concentration-dependent style, indicating a direct nonspecific toxic impact of PD against lung cells (Fig. 3). Besides, this kind of repressive effect came to be most obvious at 48 h post-IR (Fig. 3a, i). On the other hand, under several certain conditions, the radio-sensitization of PD on both kinds of cell lines was observed as absent of cellular toxicity. For example, regarding BEAS 2B, at 72 h post-IR with the PD concentration of 10 and 50 μM (Fig. 3d, h), while for A549, at 24 h post-IR with the PD concentration of 50 μM (Fig. 3j, n) or 72 h with 10 μM, 50 μM and 100 μM (Fig. 3l, p), IR-induced cell viability inhibition was significantly enhanced by PD. Thus, PD is more potent to lung cancer cells than normal epithelial cells in aggravating RT-decreased cell viability. Collectively, though PD exerts a direct toxic effect upon lung cells, it can still further attenuate the IR-induced cell viability.

According to the above, we can learn that PD treatment decreases lung cell viability in a time-dependent pattern with the most serious inhibition time point being at 48 h, it is logically speculated that long-term cell incubation may be needed to further evaluate PD’s regulatory effects on lung cells’ reproductive death after IR treatment. Therefore, the clonogenic assay of both BEAS 2B and A549 was performed. With different cell lines (BEAS 2B and A549) undergoing different concentrations of PD (50 μM and 100 μM) and exposed to different IR doses (0, 2, 4, 6, and 8GY), the clonogenic assay displayed no observable differences between each CON and Drug-treated groups (Fig. 3q, r).

### Independence of PD’s cell radiosensitivity regulation on influencing apoptosis

**Fig. 4:**
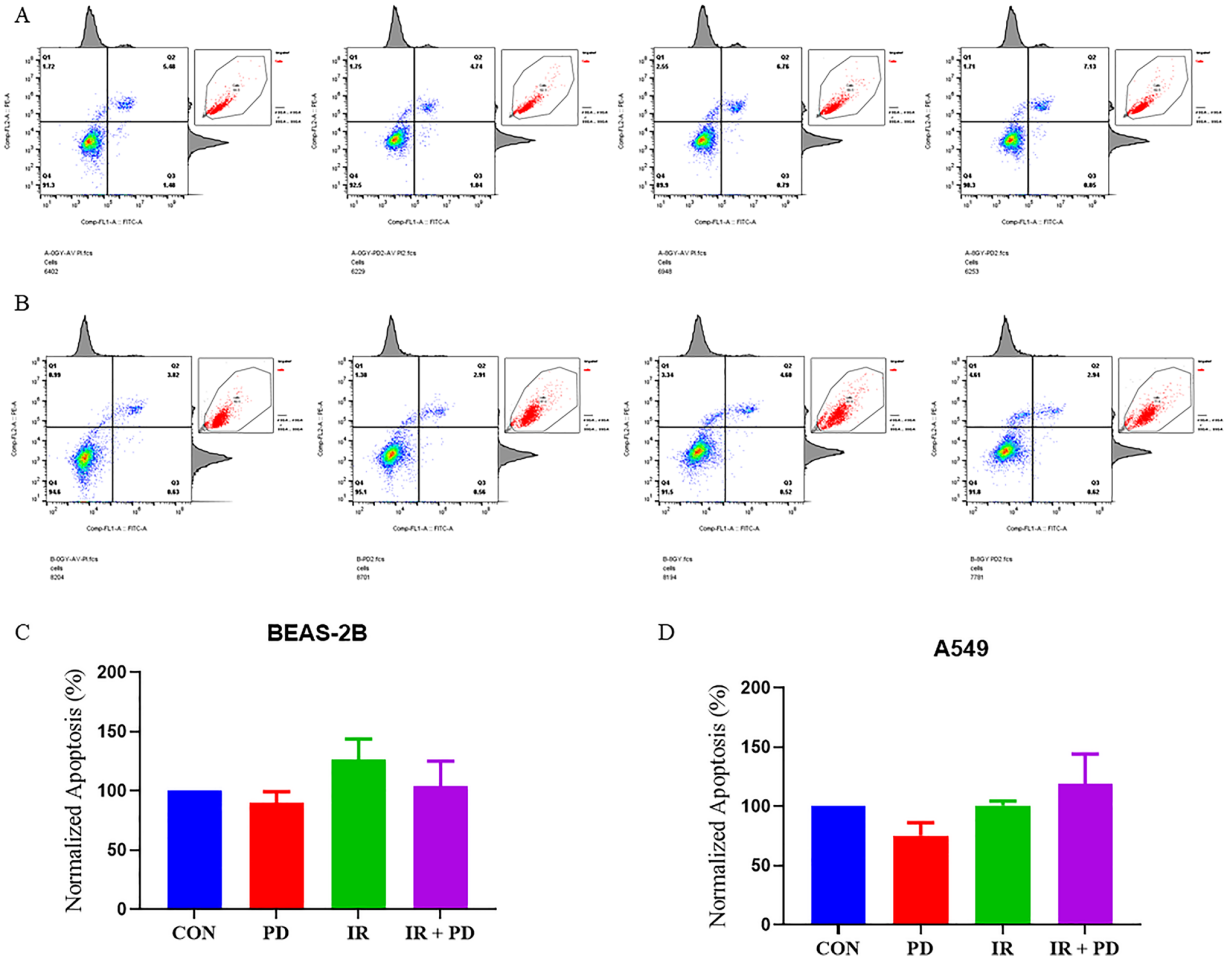
Cellular apoptosis is determined by flow cytometry. **a**, **b** Representative scatter plots of the flow cytometry results. The small panel in the up right corner of each plot depicts the distribution of the according parent population. **c**, **d** Quantitative comparison of the apoptotic rates between different treatment groups

The apoptosis of both BEAS 2B and A549 was tested 24 h after IR via flow cytometry. It was indicated that at the lower concentration, PD indistinguishably enhanced both of the two cell lines’ apoptosis ratios after IR, but at the higher concentration, it's just the opposite (Fig. 4, not all data were shown). For example, Fig. 4c, d displayed this responsive trend of BEAS 2B at 100 μM and A549 at 50 μM, respectively (Fig. 4). However, no statistical differences were detected between comparative groups, implying the probable minor role of apoptotic mechanism on PD’s potent in vivo effects on improving lung cancer radiosensitivity and alleviating radiation injuries.

### PD reduces IR-induced B cell infiltration in tumor tissue

**Fig. 5:**
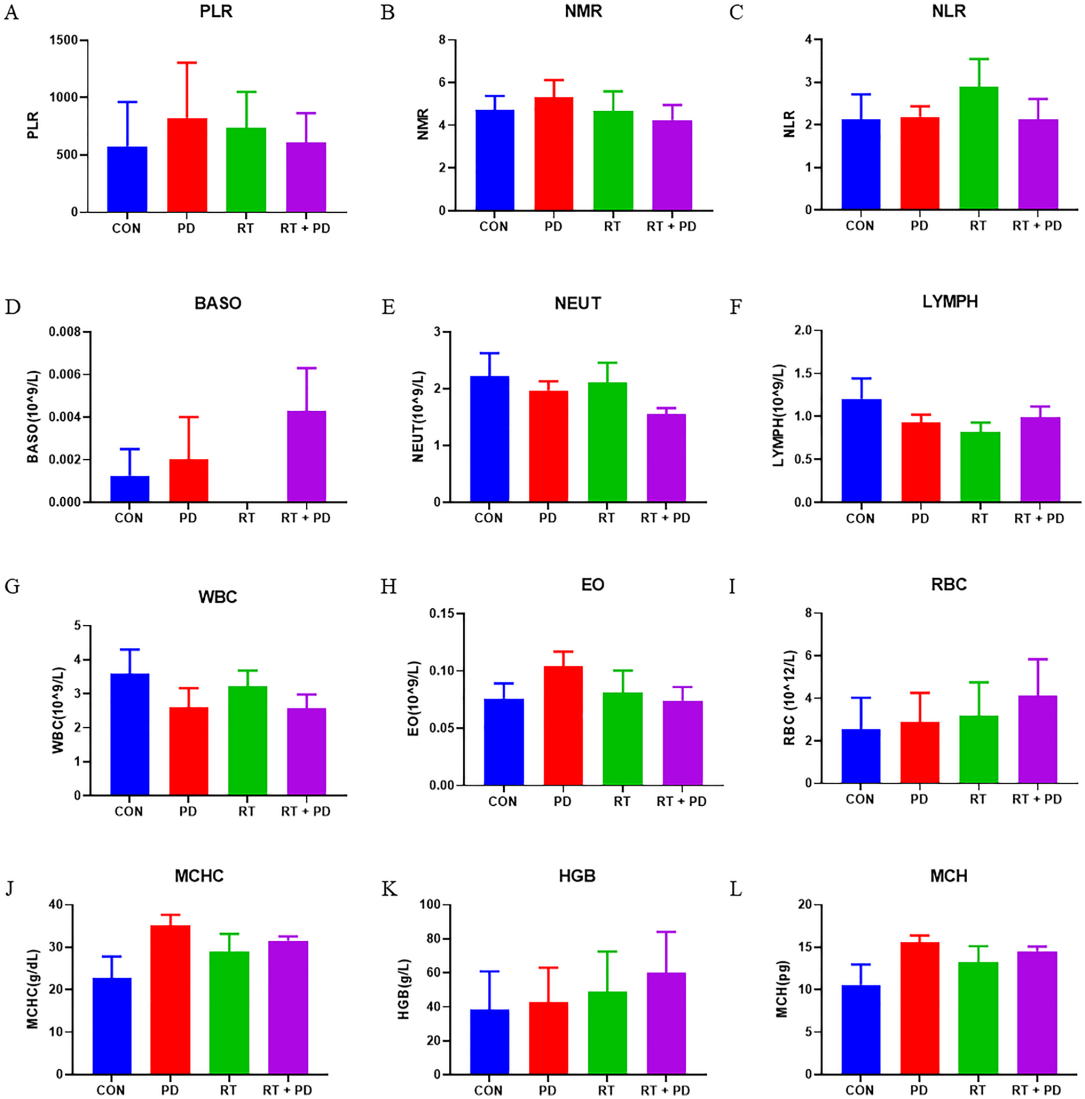
The distribution of complete peripheral blood cells was examined using an automated hematology analyzer. **a**–**c** Three of the indirect indicators calculated from the original outputs of the analyzer. **d**–**l** Parts of the hematological parameters of the mice models from different treatment groups

**Fig. 6:**
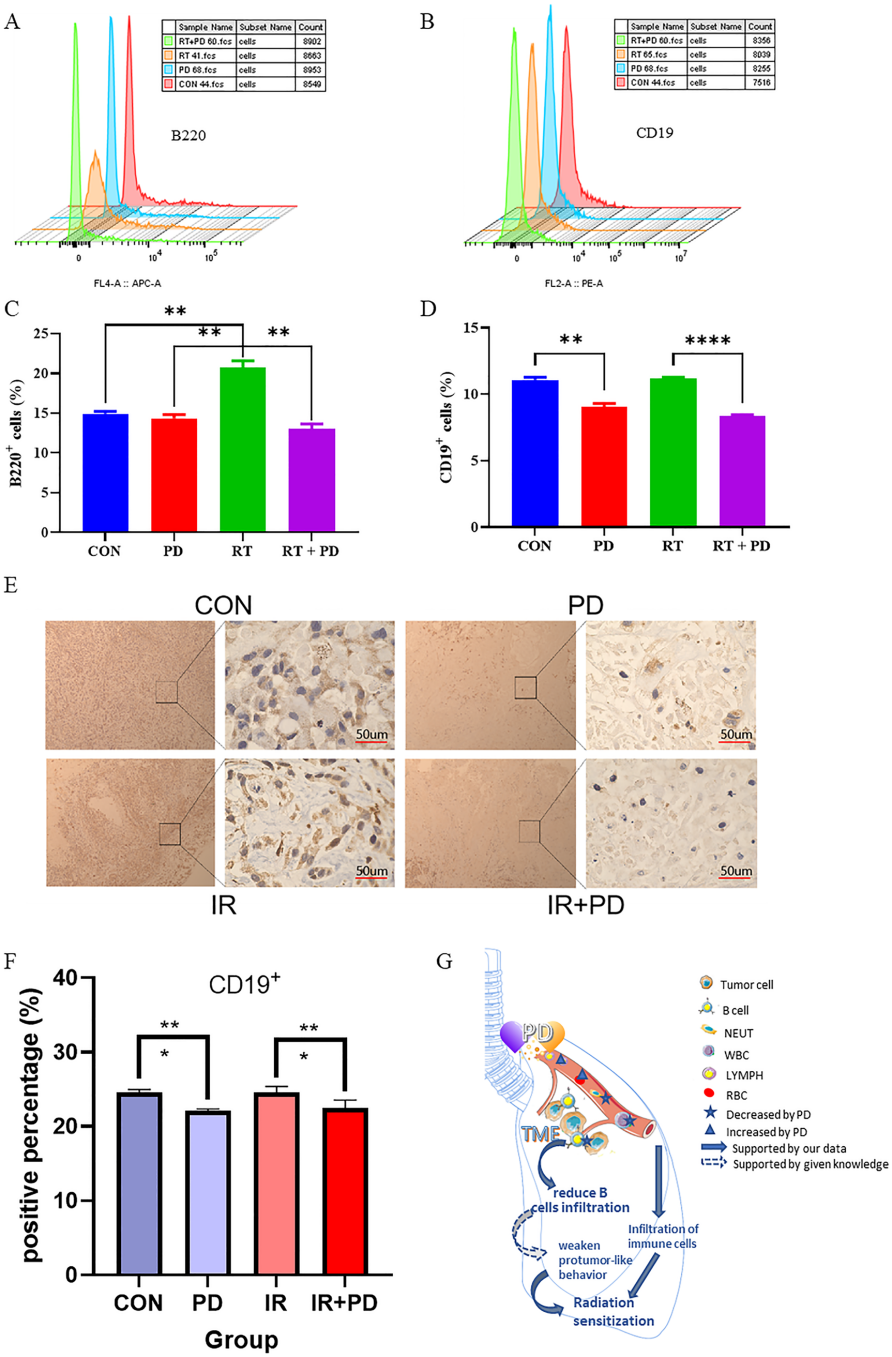
Tumor-associated B cells analysis in vivo. **a**, **b** Representative histograms of each representative treatment group in flow cytometry experiments. **c**, **d** Quantitative comparison of the B220- or CD19-positive rates between different treatment groups calculated from three independent flow cytometry tests. **e**, **f** Representative pictures and the quantitative statistical histogram for the CD19 IHC experiment for labeling B cells within the tumors. **g** Mechanism diagram for PD’s RMDF role in lung cancer radiotherapy. *p < 0.05, **p < 0.01, ****p < 0.0001

To learn more about how PD regulates radiosensitivity in lung cancer in vivo, we started by counting the peripheral blood of tumor-bearing nude mice undergoing radiotherapy. In the RT + PD group, BASO, LYMPH, RBC, MCHC, HGB, and MHC levels were greater than in the RT-only group, while NEUT and WBC were lower (Fig. 5). Moreover, we discovered that it was consistently lessened in the PD + RT group regarding parameters including PLR, NMR, and NLR when compared to the RT group (Fig. 5a–c), indicating a plausible function of immunocytes in supporting the body to modulate the lung radiosensitivity. With the above findings considered, further evaluations of the immunocytes’ function within the tumor tissue of the nude mice were carried out to get a closer study of the mechanism (Fig. 6). As the nude mice are deficient in producing T cells, B cells inside the tumors were labeled using B220 and CD19 antibodies and subjected to flow cytometry counting to reflect the B lymphocyte function. From the experiments based on both kinds of labeling, it was found that B cells were significantly recruited into the tumor tissue, whereas, PD treatment could rectify this trend markedly (Fig. 6a–d). Moreover, though the HE staining data did not present any obvious distinctions between groups (supplementary material 2), the following IHC analysis based on the CD19 labeling re-confirmed this mechanistic action of PD at the micro-histological level (Fig. 6d, e). Conclusively, these outcomes showed that PD significantly decreased RT-induced tumor B cell infiltration (Fig. 6a–e).

## Discussion

In the present research, we demonstrated that PD could radio-sensitize lung cancer and at the same time prevent radiation injuries during radiotherapy. Furthermore, we found that the mechanisms of the above effects were partly involved in the direct regulation of the targeted cell viability, but more dependent on the remolding of immune function. Thanks to its non-invasiveness, which allows for the avoidance of perioperative stress and preservation of organ structures and functioning, RT is often favored in surgical procedures in lung tumor therapy. However, this method is often limited by radioresistance, for which new attempts to develop radiosensitizers without toxifying healthy tissue haven’t stopped. As PD is a kind of natural agent with its biochemistry characteristics including the drug toxicity studied fully, its novel role in enhancing lung cancer RT effects may be more easily translated into clinical use. Thus, it's reasonable to speculate that making use of PD in combination with RT can strengthen lung cancer cells’ therapeutic response and PD can be employed as an adjuvant to RT for local disease control and reducing RT side effects.

The results of this study show that PD further diminishes the RT-treated lung tumors in a well-established mouse xenograft RT model, characterized by both lessened in vivo tumor fluorescent area and suppressed tumor volume curve. It has been reported that PD induces radiation sensitivity in both osteosarcoma cells (Luce et al. 2021) and colorectal cancer (Chen et al. 2019). In addition, a series of studies indicate that PD inhibits the proliferation, metastasis, senescence, and apoptosis of lung cancer cells, repressing tumor growth (Verma and Tiku 2022; Zou et al. 2018; Zhang et al. 2014). Considering prior research in this field as well as our findings, the current study is a helpful extension of the use of PD in oncology therapy, particularly in radiotherapy sensitization, which has the potential to improve the efficacy of radiotherapy treatment for lung cancer patients.

As we all know, an eligible radiosensitizer must not increase the sensitivity of healthy normal tissue to the undesirable radiation harm (or to a marked degree less than that of tumor tissue), in addition to being minimal toxicity. And the most persuasive approach to determine whether an agent plays a role in radioprotection is to look at animal survival in an acute radiation sickness model. According to the earlier research, PD can considerably boost the body's ability to tolerate radiation harm, as evidenced again by the current observations of animal survival (Guo, et al. 2020; Li, et al. 2018). The mechanism of its radiation protection action, as a natural plant extract that has been thoroughly explored, could include scavenging free radicals, activating antioxidant pathways, modulating immunity, boosting hematopoiesis, vascular endothelial protection, and so on (Li et al. 2013; Guo, et al. 2020; Jin et al. 2016; Pang et al. 2017). In recent years, the study of its exact mechanism has been a hot topic in the field of radiation protection research.

Another interesting finding in this study is that PD reduces cell viability after RT treatment but does not significantly influence RT-induced apoptosis in the cultured lung cell lines. This phenomenon is consistent with another finding when authors compared the effects of resveratrol and its derivatives including PD on the radiation response of breast cancer cells. Though they adopted a different cell line other than lung cancer cells and treated cells with lower concentrations of PD (5 and 25 μM), it was shown that, unlike its analog resveratrol, PD decreased the cell viability without altering apoptosis very much (Komorowska et al. 2021). However, some existing studies showed some non-consistent opinions. For example, one research has suggested that PD inhibits the growth of lung cancer cells by inducing apoptosis, for which the possible reason may be attributed to different concentrations of PD (they used less than 6 μM while ours were 50 μM and 100 μM) (Zhang et al. 2014). Despite these discrepancies, our findings are agreeable with their result in that PD is more potent in eliminating cancer cells than non-cancer cells (Zhang et al. 2014). Therefore, a similar experiment with a more detailed concentration division is needed to illustrate the PD’s concentration effects on its regulatory effects on lung cancer radiosensitivity.

As we had previously observed that PD could improve both the IR-injured peripheral hematological parameters and the immune performance, the peripheral blood cell counting parameters of the tumor-bearing mice were determined again to seek the underlying mechanisms in this study. Though no statistical differences between groups were found, we can still see the trend that PD decreases the platelet-to-lymphocyte ratio (PLR) and neutrophil-to-lymphocyte ratio (NLR) while increasing the hemoglobin to RDW ratio (HRR) (Fig. 5 and supplementary material 1). As evidenced by the past work, the higher hematologic indices of PLR and NLR, the lower patients’ overall survival in many solid tumors including lung cancers receiving treatments such as RT, while the opposite is true for HRR (Cannon et al. 2015; Bozkaya et al. 2019; Ni et al. 2014). With the above-mentioned results considered together, it is suggested that immunomodulation may play an important role in PD’s regulation of radiosensitivity in lung cancer. After investigating the tumor-infiltrating B cells by flow cytometry and IHC, we found that PD significantly alleviated the RT-surged B cells percentage in tumor tissues (Fig. 6). Previous research has demonstrated that tumor-infiltrating B lymphocytes (TIBs) can be found at all stages of lung cancer growth, implying that B cells play a key role in lung cancer progression (Dieu-Nosjean et al. 2014). In immunocompetent organisms, TIBs contribute to both humoral and cellular immunity, but their involvement in antitumor immunity is still debated (Siliņa et al. 2014). Some researchers have found that B cells can produce and maintain positive anticancer activity, whereas others have discovered that B cells may have pro-tumor behaviors as a result of their diverse immunosuppressive subtypes (Schalper, et al. 2015; Eerola et al. 2000; Suzuki et al. 2013; Kurebayashi et al. 2016). In the current study, we adopted a unique lung cancer xenograft nude mice model, which lacked a functional thymus gland, to exclude the T cell interference and focus on the role of B lymphocyte in lung tumor RT, providing unique new perspectives and evidence to uncover the mechanisms underlying both the PD’s regulation and TIBs function on lung cancer RT.

There are still several limitations in this study. First, the concentration range combined with the time course of PD administration is under-considered when detecting cell viability, apoptosis, and some other indicators. For example, different teams presented different views about how PD affects cell apoptosis with different PD concentrations (Zhang et al. 2014; Komorowska et al. 2021). Here, we showed that although multiple apoptosis results did not yield statistically different comparisons, it was still possible to find that PD was attenuating the apoptosis rate of both cell lines after 24 h but increasing it after 48 h, suggesting that its cytotoxic effects may not be mainly dependent on the apoptotic pathway. Thus, the drug's influence on whether cancerous or healthy cells may be similar here, but the drug concentrations and time both play an important part, which needs to be further investigated in detail. As a result, future research should incorporate a more comprehensive and detailed PD concentration gradient during the indicator analysis. Second, the mechanism section of this study isn't as detailed, in-depth, or concentrated as it should be. Nevertheless, it provides some vital clues for the next mechanistic exploration. Accordingly, it is suggested that a more in-depth study systematic study targeting tumor-associated B cells is needed to better understand the intrinsic connections between PD, tumor-associated B cells, and lung cancer RT in the tumor microenvironment.

All in all, by setting up an animal model of lung cancer radiotherapy, we discovered that PD can play an important role in sensitizing lung cancer toward RT and preventing RT-related radiation damage, highlighting its promising clinical implications. Furthermore, the mechanism of the above-mentioned function is based mostly on its involvement in stimulating hematopoiesis and modulating immunity in the in vivo environment, and its direct effect on target cells reflects a cytotoxic effect and impacts on RT-induced cellular viability independent of affecting apoptosis, which changes dramatically with drug concentration (Fig. 6g). To summarize, our preliminary findings show that PD could be a candidate for boosting lung cancer RT efficacy and that further research into the particular processes of lung cancer radio-sensitization and healthy tissue radio-protection is warranted.

## Supplementary Information

Below is the link to the electronic supplementary material.

Supplementary file1 Supplementary material 1. The remaining indicators obtained from the automatic peripheral complete blood counting analysis (PNG 25 KB)

Supplementary file2 Supplementary material 2. Representative HE staining pictures of tumor tissue sections from different treatment groups (PNG 712 KB)

## Data Availability

The data that support the findings of this study are available from the corresponding author (email: cjm882003@163.com), upon reasonable request.

## References

[CR1] Anjaly K, Tiku AB (2022). Caffeic acid phenethyl ester induces radiosensitization via inhibition of DNA damage repair in androgen-independent prostate cancer cells. Environ Toxicol.

[CR2] Bozkaya Y, Kurt B, Gürler F (2019). A prognostic parameter in advanced non-small cell lung cancer: the ratio of hemoglobin-to-red cell distribution width. Int J Clin Oncol.

[CR3] Cannon NA (2015). Neutrophil-lymphocyte and platelet-lymphocyte ratios as prognostic factors after stereotactic radiation therapy for early-stage non-small-cell lung cancer. J Thorac Oncol.

[CR4] Cao K (2017). Polydatin alleviated radiation-induced lung injury through activation of Sirt3 and inhibition of epithelial-mesenchymal transition. J Cell Mol Med.

[CR5] Chen Q (2019). Polydatin increases radiosensitivity by inducing apoptosis of stem cells in colorectal cancer. Int J Biol Sci.

[CR6] Delaney G (2003). A model for decision making for the use of radiotherapy in lung cancer. Lancet Oncol.

[CR7] Dieu-Nosjean MC (2014). Tertiary lymphoid structures in cancer and beyond. Trends Immunol.

[CR8] Du J (2009). Lipid-lowering effects of polydatin from *Polygonum cuspidatum* in hyperlipidemic hamsters. Phytomedicine.

[CR9] Eerola AK, Soini Y, Pääkkö P (2000). A high number of tumor-infiltrating lymphocytes are associated with a small tumor size, low tumor stage, and a favorable prognosis in operated small cell lung carcinoma. Clin Cancer Res.

[CR10] Guo J (2020). Polydatin attenuates 14.1 MeV neutron-induced injuries via regulating the apoptosis and antioxidative pathways and improving the hematopoiesis of mice. Oxid Med Cell Longev.

[CR11] Huang K (2015). Polydatin promotes Nrf2-ARE anti-oxidative pathway through activating Sirt1 to resist AGEs-induced upregulation of fibronetin and transforming growth factor-β1 in rat glomerular messangial cells. Mol Cell Endocrinol.

[CR12] Jin J (2016). Evaluation of both free radical scavenging capacity and antioxidative damage effect of polydatin. Adv Exp Med Biol.

[CR13] Komorowska D (2021). Comparison of the effects of resveratrol and its derivatives on the radiation response of MCF-7 breast cancer cells. Int J Mol Sci.

[CR14] Kurebayashi Y (2016). Comprehensive immune profiling of lung adenocarcinomas reveals four immunosubtypes with plasma cell subtype a negative indicator. Cancer Immunol Res.

[CR15] Li XH (2013). Protective effects of polydatin on septic lung injury in mice via upregulation of HO-1. Mediat Inflamm.

[CR16] Li L (2018). Protective effect of polydatin on radiation-induced injury of intestinal epithelial and endothelial cells. Biosci Rep.

[CR17] Luce A (2021). Polydatin induces differentiation and radiation sensitivity in human osteosarcoma cells and parallel secretion through lipid metabolite secretion. Oxid Med Cell Longev.

[CR18] Miao Q (2012). Polydatin attenuates hypoxic pulmonary hypertension and reverses remodeling through protein kinase C mechanisms. Int J Mol Sci.

[CR19] Mikulski D, Molski M (2010). Quantitative structure-antioxidant activity relationship of trans-resveratrol oligomers, trans-4,4'-dihydroxystilbene dimer, trans-resveratrol-3-O-glucuronide, glucosides: trans-piceid, cis-piceid, trans-astringin and trans-resveratrol-4′-O-beta-d-glucopyranoside. Eur J Med Chem.

[CR20] Miyake M (2019). Dual benefit of supplementary oral 5-aminolevulinic acid to pelvic radiotherapy in a syngenic prostate cancer model. Prostate.

[CR21] Ni XJ (2014). An elevated peripheral blood lymphocyte-to-monocyte ratio predicts favorable response and prognosis in locally advanced breast cancer following neoadjuvant chemotherapy. PLoS ONE.

[CR22] Pang N (2017). Polydatin prevents methylglyoxal-induced apoptosis through reducing oxidative stress and improving mitochondrial function in human umbilical vein endothelial cells. Oxid Med Cell Longev.

[CR23] Schalper KA et al (2015) Objective measurement and clinical significance of TILs in non-small cell lung cancer. J Natl Cancer Inst 107(3):dju435 10.1093/jnci/dju435 PMC4565530 25650315

[CR24] Shiyu S (2011). Polydatin up-regulates Clara cell secretory protein to suppress phospholipase A2 of lung induced by LPS in vivo and in vitro. BMC Cell Biol.

[CR25] Siliņa K (2014). Manipulation of tumour-infiltrating B cells and tertiary lymphoid structures: a novel anti-cancer treatment avenue?. Cancer Immunol Immunother.

[CR26] Suzuki K (2013). Clinical impact of immune microenvironment in stage I lung adenocarcinoma: tumor interleukin-12 receptor β2 (IL-12Rβ2), IL-7R, and stromal FoxP3/CD3 ratio are independent predictors of recurrence. J Clin Oncol.

[CR27] Thai AA (2021). Lung cancer. Lancet.

[CR28] Verma N, Tiku AB (2022). Polydatin-induced direct and bystander effects in A549 lung cancer cell line. Nutr Cancer.

[CR29] Yang B (2013). Polydatin attenuated food allergy via store-operated calcium channels in mast cell. World J Gastroenterol.

[CR30] Zhang Y (2014). Polydatin inhibits growth of lung cancer cells by inducing apoptosis and causing cell cycle arrest. Oncol Lett.

[CR31] Zou J (2018). Polydatin suppresses proliferation and metastasis of non-small cell lung cancer cells by inhibiting NLRP3 inflammasome activation via NF-κB pathway. Biomed Pharmacother.

