# Supplementary figures and images for: Polydatin radiosensitizes lung cancer while preventing radiation injuries by modulating tumor-infiltrating B cells
Source: J Cancer Res Clin Oncol. 2023 May 23;149(12):9529–42. doi: 10.1007/s00432-023-04762-7 (PMC10423126; doi:10.1007/s00432-023-04762-7)

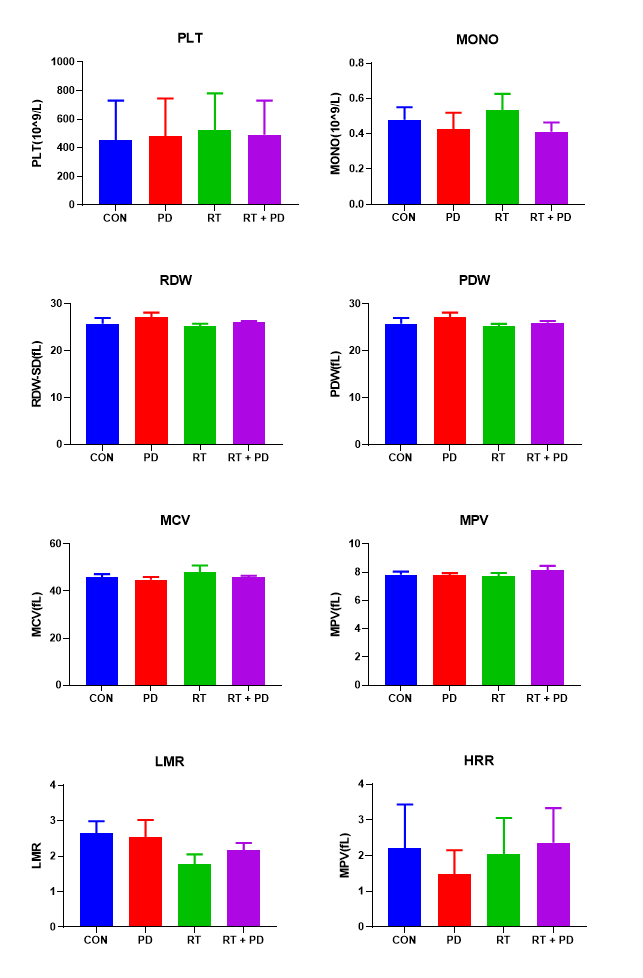

Supplement: Supplementary file 1 — Supplementary file1 Supplementary material 1. The remaining indicators obtained from the automatic peripheral complete blood counting analysis (PNG 25 KB) [file 432_2023_4762_MOESM1_ESM.png]

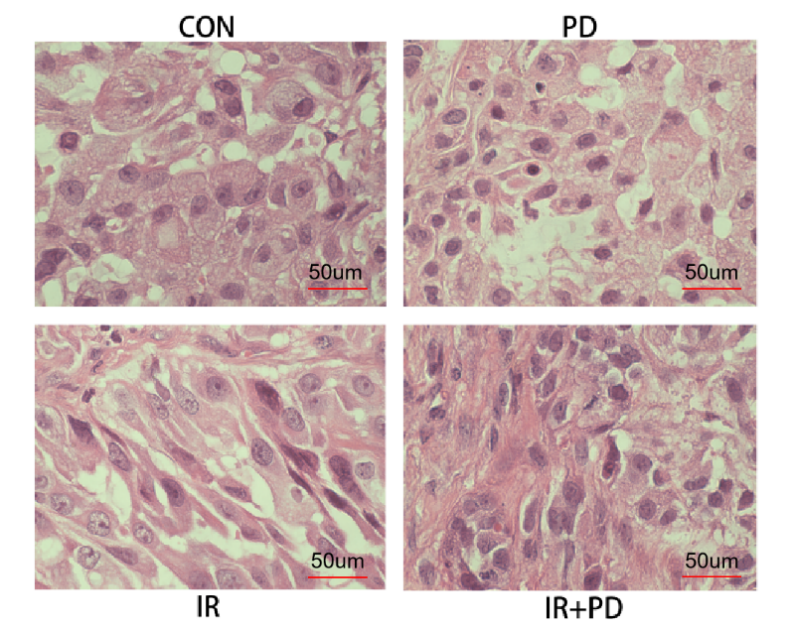

Supplement: Supplementary file 2 — Supplementary file2 Supplementary material 2. Representative HE staining pictures of tumor tissue sections from different treatment groups (PNG 712 KB) [file 432_2023_4762_MOESM2_ESM.png]
